# Long non‐coding RNAs mediate fish gene expression in response to ocean acidification

**DOI:** 10.1111/eva.13655

**Published:** 2024-02-14

**Authors:** Jingliang Kang, Arthur Chung, Sneha Suresh, Lucrezia C. Bonzi, Jade M. Sourisse, Sandra Ramirez‐Calero, Daniele Romeo, Natalia Petit‐Marty, Cinta Pegueroles, Celia Schunter

**Affiliations:** ^1^ Swire Institute of Marine Science, School of Biological Sciences The University of Hong Kong Pokfulam Hong Kong SAR; ^2^ Department of Genetics, Microbiology and Statistics, Institute for Research on Biodiversity (IRBio) University of Barcelona Barcelona Spain; ^3^ State Key Laboratory of Marine Pollution and Department of Chemistry City University of Hong Kong Hong Kong SAR China

**Keywords:** annotation, environmental change, epigenetic regulation, lncRNAs, ocean acidification, RNA sequencing

## Abstract

The majority of the transcribed genome does not have coding potential but these non‐coding transcripts play crucial roles in transcriptional and post‐transcriptional regulation of protein‐coding genes. Regulation of gene expression is important in shaping an organism's response to environmental changes, ultimately impacting their survival and persistence as population or species face global change. However, the roles of long non‐coding RNAs (lncRNAs), when confronted with environmental changes, remain largely unclear. To explore the potential role of lncRNAs in fish exposed to ocean acidification (OA), we analyzed publicly available brain RNA‐seq data from a coral reef fish *Acanthochromis polyacanthus*. We annotated the lncRNAs in its genome and examined the expression changes of intergenic lncRNAs (lincRNAs) between *A. polyacanthus* samples from a natural CO_2_ seep and a nearby control site. We identified 4728 lncRNAs, including 3272 lincRNAs in this species. Remarkably, 93.03% of these lincRNAs were species‐specific. Among the 125 highly expressed lincRNAs and 403 differentially expressed lincRNAs in response to elevated CO_2_, we observed that lincRNAs were either neighboring or potentially trans‐regulating differentially expressed coding genes associated with pH regulation, neural signal transduction, and ion transport, which are known to be important in the response to OA in fish. In summary, lncRNAs may facilitate fish acclimation and mediate the responses of fish to OA by modulating the expression of crucial coding genes, which offers insight into the regulatory mechanisms underlying fish responses to environmental changes.

## 1 INTRODUCTION

Among pervasive genomic regions that can be transcribed, some encode long non‐coding RNAs (lncRNAs), defined as RNAs longer than 200 nucleotides that are not translated into functional proteins. LncRNAs represent a highly heterogeneous class of transcripts mainly transcribed by RNA polymerase II and, usually, inefficiently spliced (Statello et al., 2021). Once thought to be merely products of transcriptional noise or spurious transcription (Struhl, 2007), lncRNAs have now been regarded as key players in various biological processes, including DNA repair, cell proliferation, and embryonic development (Fernandes et al., 2019; Li et al., 2019; Vance & Ponting, 2014). LncRNAs are involved in chromatin modifications, pre‐transcription, transcription, and post‐transcription through regulation of the associated gene expression (Gardini & Shiekhattar, 2015; Kornfeld & Brüning, 2014; Necsulea et al., 2014; Statello et al., 2021; Ulitsky, 2016; Zhu et al., 2013). In addition, lncRNAs may lead to gene dysfunction and can be used as biomarkers of certain diseases (Beck et al., 2018; Fernandes et al., 2019; Jiang et al., 2016).

Despite the importance and growing interest in lncRNAs as well as the availability of RNA sequencing data in many species, lncRNAs pose challenges for identification due to their lower conservation compared to protein‐coding sequences, resulting from their rapid turnover (Lopez‐Ezquerra et al., 2017; Pegueroles et al., 2019), as well as their cell and tissue specificity (Cabili et al., 2015; Derrien et al., 2012). To date, several bioinformatic tools have been developed to detect lncRNAs by computing coding potential score (CPS) of transcripts, either by using sequence alignments (alignment‐dependent) or detecting intrinsic features of the input RNA sequences (alignment‐free). In the alignment‐dependent methods, such as PhyloCSF (Lin et al., 2011) and CPC (Kong et al., 2007), sequences are either aligned between species or to protein databases, which may be biased toward misclassifying species‐specific or lowly conserved coding and non‐coding transcripts. In contrast, although alignment‐free methods like CPAT (Wang et al., 2013) and FEELnc (Wucher et al., 2017) are useful to discriminate species‐specific lncRNA, these tools use different intrinsic features (such as the length and integrity of the longest open reading frame) of the input RNA sequences, which can result in differences in detecting lncRNAs. Nevertheless, most studies related to lncRNA identification used only one method (Boltaña et al., 2016; Dettleff et al., 2020; Mu et al., 2016; Paneru et al., 2018; Quan, Chen, et al., 2020; Ren et al., 2020). Hence, integrating both alignment‐dependent and alignment‐free approaches could offer a reliable path to detect conserved and species‐specific lncRNAs.

Environmental perturbations produce molecular responses in organisms needed to maintain cellular homeostasis and function, and lncRNAs may play a role in some of these responses by regulating gene expression (Paneru et al., 2018; Sarangdhar et al., 2018) through cis‐ and trans‐regulation (Gil & Ulitsky, 2020; Luo et al., 2019; Nadal‐Ribelles et al., 2014; Quan, Chen, et al., 2020). A large proportion of lncRNAs with functional implications are in fact cis‐regulatory and hence affect the regulation of the neighboring protein‐coding genes allowing for plasticity in gene networks across time (Engreitz et al., 2016; Gil & Ulitsky, 2020). In the model‐plant genus *Arabidopsis*, for instance, a lncRNA tightly controls the expression of highly conserved transcription factors that promote cold tolerance in many plant species (Kindgren et al., 2018). In vertebrates, such as fish, lncRNAs identified in rainbow trout (*Oncorhynchus mykiss*) can potentially mediate regulation of the heat stress response (Quan, Kang, et al., 2020) and one lncRNA affects the mucosal immunity of turbot (*Scophthalmus maximus*) in response to bacterial infection (Yang et al., 2020). The involvement of lncRNAs in the regulation of the response to environmental change reveals its importance as a potential regulator of (phenotypic) plasticity and in turn adaptive potential in rapidly changing environments. Hence, there is a need for more studies to clarify the role of lncRNAs in the molecular response of species to changing environment.

At present, anthropogenic activities have caused various environmental changes, including ocean acidification (OA) with a predicted change in ocean pH to pH 7.7 by the end of this century (Pörtner et al., 2022). A coral reef fish *Acanthochromis polyacanthus*, a common coral reef fish in the Western Pacific, has extensively been studied for fish acclimation processes to OA. Generous RNA sequencing has been performed on this species, especially on the brain tissue (Bernal et al., 2022; Kang et al., 2022; Schunter et al., 2016, 2021) because altered function of GABA_A_ neurotransmitter receptors is thought to be responsible for many behavioral changes observed in fish exposed to OA (Heuer & Grosell, 2014; Nilsson et al., 2012). The impact of OA on this species has been linked to alterations in the expression of coding genes associated with circadian rhythm, intracellular pH regulation, ion transport, and immune response (Kang et al., 2022; Schunter et al., 2016, 2018). However, the potential influence of lncRNAs in responding to OA in this species remains unexplored. To evaluate the involvement of lncRNAs on gene expression in fish exposed to OA, we combined the expression of lncRNAs and coding genes in *A. polyacanthus* to examine the transcriptional and epigenetic factors that shape the organism's biological response to OA. Meanwhile, we aim to offer a comprehensive roadmap for the identification of lncRNAs, guiding ecologists and environmental scientists to utilize their RNA‐seq data to investigate lncRNA regulation in response to environmental shifts.

## 2 METHODS

### 2.1 LncRNAs identification

**FIGURE 1:**
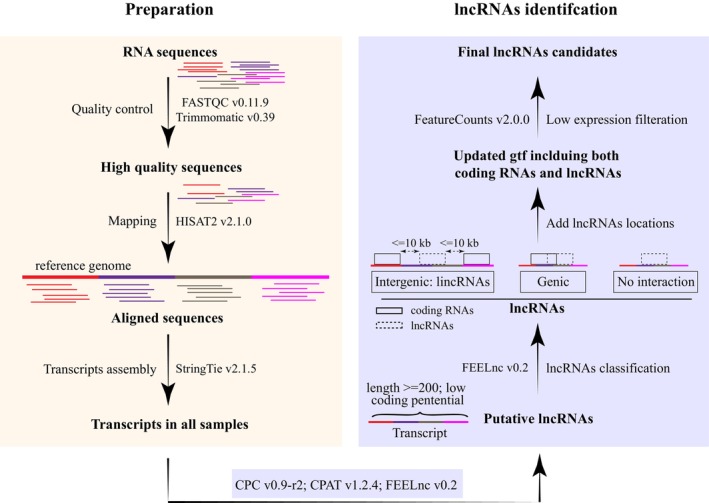
Flowchart illustrating the process for long non‐coding RNA (lncRNA) identification in *Acanthochromis polyacanthus* genome. In yellow are the steps for the preparation of input transcripts for three tools (CPC, CPAT, and FEELnc) to identify putative lncRNAs, while in blue are the lncRNAs identification for the creation of the final high‐confidence lncRNA set. Software and versions are stated for each step.

Due to the lack of lncRNA annotations in the *Acanthochromis polyacanthus* genome, we applied a pipeline (Figure 1) integrating both alignment‐dependent and alignment‐free methods to identify lncRNAs in *A. polyacanthus* genome. For this, 226 RNA‐seq *A. polyacanthus* individuals (details in Table S1) sampled in wild and laboratory with different CO_2_ levels were compiled from published studies (Kang et al., 2022; Schunter et al., 2016, 2018) and one unpublished study (NCBI bioproject: PRJNA658203) investigating the effect of ocean acidification (OA) on the brain gene expression of *A. polyacanthus*. Whole brain tissue of these 226 *A. polyacanthus* individuals were dissected out, and RNAeasy kits (Qiagen) were used to extract RNA for sequencing via polyadenylated (poly‐A^+^) selection (Kang et al., 2022; Schunter et al., 2016, 2018) on the Illumina platform. This allows for a comprehensive annotation across the genome of lncRNAs associated with the exposure to OA in laboratory and natural settings. To identify a reliable set of lncRNAs across the genome (Figure 1), the raw reads quality was first assessed with FASTQC v0.11.9, and then adapter sequences and low‐quality sections of reads were removed by Trimmomatic v0.39 (Bolger et al., 2014) using the following parameters: “ILLUMINACLIP:2:30:10 LEADING:4 TRAILING:3 SLIDINGWINDOW:4:20 MINLEN:40”. The resulting clean reads were aligned to the reference genome of *A. polyacanthus* (NCBI database, accession number: ASM210954v1) using a fast and sensitive alignment program HISAT2 v2.1.0 (Kim et al., 2019) with default parameters and “–*known‐splicesite‐infile*” to provide known splice sites. StringTie v2.1.5 (Pertea et al., 2016) was then applied to assemble transcripts. All resulting transcripts across all of the 226 fish samples were merged for a unified set of non‐redundant transcripts using the transcript merge mode (−‐merge) of StringTie (Pertea et al., 2016).

To estimate the coding potential of all transcripts, we extracted the transcript sequences from the *A. polyacanthus* genome sequence using GffRead v0.12.7 (Pertea & Pertea, 2020) with the parameters “*‐w, ‐g*”. Three tools, including one alignment‐dependent (CPC) and two alignment‐free methods (CPAT and FEELnc), were used to calculate the coding potential of all transcripts. CPC v0.9‐r2 (Kong et al., 2007) was used with default parameters to estimate coding potential based on sequence similarity between transcripts and Uniref90 protein database, and the transcripts with a coding potential score <0 were considered as putative lncRNAs. For the two alignment‐free tools, CPAT v1.2.4 (Wang et al., 2013) was run with default parameters using the coding mRNA sequences of zebrafish as training model, and transcripts with a coding probability <0.38 suggested by CPAT (Wang et al., 2013) were considered as putative lncRNAs. The second alignment‐free tool FEELnc v0.2 (Wucher et al., 2017), which annotates lncRNAs based on a Random Forest model trained with general features such as multi k‐mer frequencies and relaxed open reading frames, was run with FEELnc_filter.pl to filter out spurious coding transcripts using “‐b transcript_biotype=protein_coding, –monoex=‐1”, and then with default parameters FEELnc_codpot.pl to compute the coding potential score for each transcript by setting the mode of the lncRNA simulation to intergenic. The putative lncRNAs were then identified based on the best coding potential cut‐off of receiver operating characteristic curve plot (Sing et al., 2005). For all three methods, only genes with all transcripts identified as non‐coding were retained for further analyses.

For a more conservative approach in avoiding spurious lncRNA annotations, we only retained the transcripts that were identified as lncRNAs by at least two tools. Using a window size of 100 kilobase (kb) suggested in previous studies (Chen et al., 2017; Hosmani et al., 2019; Sarropoulos et al., 2019; Ye et al., 2022), these putative lncRNAs were categorized by computing interactions with their proximal coding transcripts using default parameters of FEELnc_classifier.pl (Wucher et al., 2017). A lncRNA is classified as “intergenic” (i.e., lincRNA) if all transcripts of this lncRNA have no location overlap with any neighboring coding genes. Due to the fact that intergenic lncRNA (lincRNA) gene expression patterns, sequence conservation and perturbation outcomes are easier to interpret than those of transcripts from other lncRNAs, for instance, genic lncRNAs which overlap with coding genes in the genomic location (Ulitsky & Bartel, 2013), only lncRNA genes classified as intergenic were used in further analyses to investigate their potential involvement in the response to ocean acidification in fish.

The expression levels of the putative lincRNAs and mRNAs were quantified using FeatureCounts v2.0.0 (Liao et al., 2014) allowing for multi‐mapped reads to be counted fractionally. The transcripts were retained if they were expressed (reads number >0) in at least 75% (170 individuals) of 226 individuals to remove lowly expressed transcripts. The read numbers of remaining transcripts were normalized using DESeq2 v1.32.0 (Love et al., 2014), and transcripts with a normalized reads number ≥1 in at least 90% individuals were remained to obtain the final candidate lincRNAs. This provided us with a final set of high‐confidence lincRNAs in both location and expression. LincRNAs with more than 500 normalized reads were considered as highly expressed lincRNAs to investigate possible functional implications of candidate lincRNAs.

### 2.2 Assessment of lincRNAs conservation across *A. polyacanthus* and other fish species

To evaluate the conservation of lincRNAs found in *A. polyacanthus* across fish species, all *A. polyacanthus* lincRNA sequences were used as a query to search against the lncRNAs sequences of RNAcentral release 23 (https://rnacentral.org/) using the default parameters of BLASTn. The hits were selected by e‐value, and only the best scoring hits were retained if the subject sequences were lncRNAs from fish species. To identify the orthologous lincRNAs across *A. polyacanthus* and other fish species, the first step was then to acquire the lincRNAs of these species for which the best‐scoring lncRNA hits were obtained. For this, we downloaded the genome annotation information (gtf file) for each species from Ensembl Release 110 (July 2023, https://asia.ensembl.org/info/data/ftp/index.html). We extracted lncRNA annotations from transcripts tagged as “lncRNA” or “lincRNA” and coding gene annotations from transcripts tagged as “protein_coding”. Subsequently, we used FEELnc_classifier.pl from FEELnc v0.2 (Wucher et al., 2017) with default parameters, with the lncRNA annotation gtf file and the coding gene annotation gtf file as inputs to identify lincRNAs for each species. Following this, we applied a reciprocal method between *A. polyacanthus* and each fish species using BLASTn with an e‐value cut‐off of 1e‐3 as applied in (Pegueroles et al., 2019) to identify orthologous lincRNAs between *A. polyacanthus* and other fish species. In the reciprocal method (Kern et al., 2018), we required that the best scoring hit (measured by e‐value) aligning *A. polyacanthus* to species A, can be matched to the best scoring hit aligning in the reverse direction (from species A to *A. polyacanthus*). Orthologous lincRNAs were accepted if lincRNAs from any mapped fish species showed reciprocal best hits to the *A. polyacanthus* lincRNAs.

To verify if orthologous lincRNAs in *A. polyacanthus* have the same neighboring coding genes across different fish species, we annotated neighboring coding genes of orthologous lincRNAs by mapping their longest transcript protein sequences to the Swiss‐Prot database (560,823 proteins, release‐2019‐11) in UniProt (release‐2019‐11) using DIAMOND blastp v0.9.22 (Buchfink et al., 2015) by default parameters. Orthologous lincRNAs in *A. polyacanthus* were considered to share the same neighboring coding genes if at least one fish species had orthologous lincRNAs whose neighboring coding genes were annotated to the same gene name as *A. polyacanthus*.

### 2.3 Expression patterns of lincRNAs in fish living in CO_2_ seep

Using the brain RNA sequencing data from wild *A. polyacanthus* individuals collected in a CO_2_ seep and its nearby control sites with ambient CO_2_ level at Papua New Guinea (Kang et al., 2022; NCBI Bioproject PRJNA691990), we evaluate the expression changes of lincRNAs in different environmental conditions. Seven individuals were collected from a coral reef situated in a CO_2_ seep with naturally bubbling CO_2_ which induced ocean acidification (pH = 7.77, *p*CO_2_ = 843 μatm) close to the predicted CO_2_ levels for the end of this century (Schmidt, 2022). Eleven individuals were sampled from an adjacent control reef with ambient CO_2_ level (pH = 8.01, *p*CO_2_ = 443 μatm) approximately 500 m away from the CO_2_ seep. There was no significant difference in temperature and salinity between the CO_2_ seep and the control site (Fabricius et al., 2011) allowing for the evaluation of effects of long‐term ocean acidification conditions on fish.

To evaluate differential gene expression of lincRNAs as well as their potential impacts on coding genes between *A. polyacanthus* individuals from CO_2_ seep and the control site, we performed differential expression analysis on lincRNAs and coding genes using DESeq2 v1.34 (Love et al., 2014). Between individuals from control and CO_2_ seep, lincRNAs and coding genes were considered as differentially expressed (DE) with an FDR adjusted *p*‐value ≤ 0.05, and the average of the normalized count values (basemean) ≥10 as well as Log2FoldChange ≥0.3. A principal component analysis (PCA) was performed using the log 2‐fold normalized expression of the samples from the two different CO_2_ level sites.

To identify lincRNAs and coding gene co‐expression modules with significant correlation with CO_2_ levels, a weighted gene co‐expression network analysis (WGCNA) (Langfelder & Horvath, 2008) was also applied on the same fish individuals. The co‐expression similarity of gene modules was obtained by selecting a *signed* network adjacency type with a soft thresholding power of 10 calculated according to the scale‐free topology criteria. We established the expression similarity between nodes of genes that are co‐expressed by calculating the topological overlap measure (TOM). These co‐expression networks were grouped into different colored modules based on their eigengenes values. Pearson correlation was then calculated to evaluate the correlation between gene modules and samples from control and CO_2_ seep. The coding genes and lincRNAs within the significantly correlated gene modules (*p* < 0.01) were selected in the following analysis.

### 2.4 Prediction of potential cis‐ and trans‐acting lincRNAs

To investigate the potential role of cis‐acting lincRNAs in the response to ocean acidification (OA), we focused on (a) neighboring coding genes of highly expressed lincRNAs, (b) differentially expressed (DE) neighboring coding genes of DE lincRNAs, and (c) neighboring coding genes co‐expressed with lincRNAs in the same WGCNA module. To investigate the potential role of trans‐acting lincRNAs (except for the lincRNAs that have neighboring coding genes) in the response to OA, we estimated the association relationship for (d) DE lincRNAs and DE coding genes, and (e) lincRNAs and coding genes within the same WGCNA module using Spearman's correlation test (Tsai et al., 2021) in R version 3.6.3 based on the normalized reads number per lincRNA‐coding gene pair. The lincRNAs that showed Spearman's correlation coefficient |rho| ≥ 0.9 and *p*‐value ≤ 0.01 with a coding gene, were considered as potential trans‐acting lincRNAs. Such correlated coding genes were used for functional enrichment analysis. For all subsets, functional enrichment analyses were performed in OmicsBox v2.0.36 (BioBam Bioinformatics, 2019) with all the annotated genes in *A. polyacanthus* genome as reference.

## 3 RESULTS

### 3.1 LncRNAs identification in *Acanthochromis polyacanthus*

**FIGURE 2:**
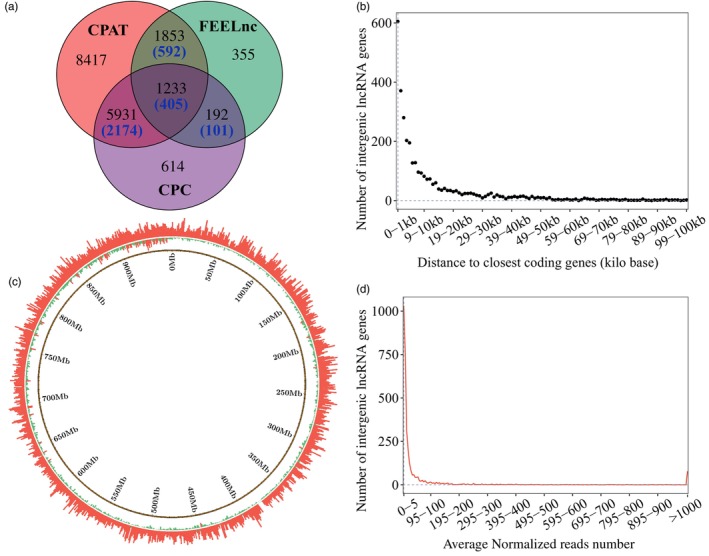
Intergenic lncRNA genes (lincRNAs) along the *Acanthochromis polyacanthus* genome. (a) Venn diagram of putative lncRNAs identified by CPAT, CPC, and FEELnc programs. Blue numbers in brackets indicate overlapping lincRNAs among the total lncRNAs. (b) Distance ranges between the identified 3272 lincRNAs and their neighboring coding genes. (c) Distribution of lincRNAs (first outer circle) and coding genes (second outer circle) along the whole genome (concatenating all scaffolds together from longest to shortest). Genome sliding windows are indicated in the inner black circle. Bar height and color are indicative of the number of genes found in each 1 Mb sliding window, with red and green bars indicating more and less than 10 lincRNAs or coding genes per window, respectively. (d) LincRNAs levels of expression across different expression level ranges.

Brain *Acanthochromis polyacanthus* RNA sequencing samples had on average 32.7 million high‐quality paired‐end reads (Table S1) and on average, 87.5% of these reads mapped to the reference genome (Table S2). The assembly steps resulted in a total of 116,237 transcripts across the genome. We used different algorithms to predict the coding potential score (CPS) of these transcripts. This resulted in 29,127 non‐coding transcripts with CPAT, 28,865 with CPC, and 11,897 with FEELnc, belonging to 17,434, 7970, and 3633 putative long non‐coding RNA genes (lncRNAs), respectively. Of these, 9209 putative lncRNAs were identified by at least two programs (Figure 2a). After filtering out lowly expressed putative lncRNAs, we obtained a final set of 4728 lncRNAs, consisting of 1313 genic, 3272 intergenic, and 143 non‐neighboring lncRNAs.

**FIGURE 3:**
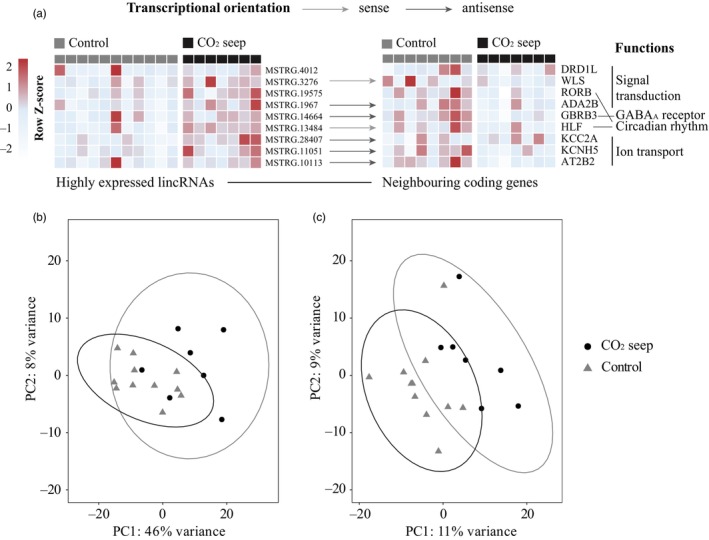
Highly expressed lincRNAs (left) in *Acanthochromis polyacanthus* and their neighboring coding genes (a), black means the *A. polyacanthus* individuals from CO_2_ seep, and grey means individuals from the control site. Heatmap was created based on the transcripts per million of lincRNAs or neighboring coding genes and scaled by row. Columns with dark red indicated that the lincRNAs or neighboring coding genes were highly expressed. Principal component analyses (PCAs) of log2‐fold normalized expression values of (b) lincRNA and coding genes or (c) lincRNAs only in the brain of wild collected *A. polyacanthus* from CO_2_ seep (circles) and the control site (triangles). Ellipse areas represent a 95% confidence level. The arrowed lines indicate the transcriptional orientation (sense or antisense) of lincRNAs in relation to their neighboring coding genes, while the absence of an arrow suggests an unknown orientation. The term “sense” refers to a shared transcriptional direction between the lincRNA and its adjacent coding gene, whereas “antisense” denotes an opposing transcriptional orientation.

The intergenic lncRNA genes (lincRNAs; Table S3) are significantly shorter in length than coding genes (two‐sample Wilcoxon rank sum test, *p* < 2.2e‐16) with 1386 lincRNAs with a length less than 400 nucleotides (Figure S1a). Of all intergenic lncRNA genes (Table S3), 2143 (65.5%) lincRNAs are monoexonic, while 1129 (34.5%) lincRNAs have at least two exons (Figure S1b). In comparison, 30,282 coding genes (88.6%) have at least two exons, which are significantly more than lincRNAs (two‐sample Wilcoxon rank sum test, *p* < 2.2e‐16). The GC content (43.1%) of lincRNAs is significantly lower than coding genes (50.0%, two‐sample Wilcoxon rank sum test, *p* < 2.2e‐16, Figure S1c). Of these 3272 lincRNA, 354 are antisense, 243 are sense lincRNAs, and the rest are strand‐unknown lincRNAs (Table S3). Among the 354 antisense lincRNAs, four lincRNAs (MSTRG.12220, MSTRG.40264, MSTRG.36665, and MSTRG.10872) contain both convergent and divergent transcripts, while 146 and 204 only contained divergent or convergent transcripts, respectively (Figure S2). LincRNAs were dispersed throughout the whole genome (Figure 2c); however, the overall lincRNAs density was significantly lower than in protein‐coding genes (two‐sample Wilcoxon rank sum test, *p* < 2.2e‐16). Among 955 genomic sliding windows of 1 Mb in the *A. polyacanthus* genome, 829 regions included more than one lincRNA and 53 regions included more than 10 lincRNAs. Protein coding genes, by contrast, displayed a higher density (934 regions with more than 10 coding genes). Most lincRNAs exhibited expression with less than 500 normalized reads, and only 125 lincRNAs had elevated expression levels (average normalized reads >500; Figure 2d; Table S4). The neighboring coding genes of the 125 most expressed lincRNAs were involved in signal transduction, GABA‐A receptor, circadian rhythm, and ion transport (Figure 3a; Table S4). The highest expressed lincRNA (MSTRG.9528; expression >190,000 normalized reads) neighbors the coding gene peripherin‐2 (PRPH2).

### 3.2 Conservation of lincRNAs between *A. polyacanthus* and other fish species

Among the 3272 lincRNAs identified in *A. polyacanthus*, 558 lincRNAs were successfully mapped to lncRNA sequences in RNAcentral using BLASTn, of which 551 were mapped to lncRNAs of 34 different fish species (Table S5). Notably, 96 of these 551 lincRNAs (Table S6) were mapped to lncRNAs of the Nile tilapia (*Oreochromis niloticus*), which was the fish species with the largest number of *A. polyacanthus* lincRNAs mapped among the 34 fish species.

To detect the orthologous lincRNAs across *A. polyacanthus* and the 34 mapped fish species, we first identified the lincRNAs for the 34 fish species. We found that Atlantic salmon (*Salmo salar*) had the highest number of lincRNAs (22,190 lincRNAs, Table S7), while the live sharksucker (*Echeneis naucrates*) had the lowest (236 lincRNAs). Using a reciprocal method based on the lincRNA sequences of *A. polyacanthus* and the 34 fish species, we identified that 228 lincRNAs were orthologous between *A. polyacanthus* and at least one of the 34 fish species (Table S8). Similarly, *A. polyacanthus* shared the greatest number of orthologous lincRNAs (58 lincRNAs) with the Nile tilapia (*Oreochromis niloticus*). On the other hand, *A. polyacanthus* shared only a single orthologous lincRNA with the inshore hagfish (*Eptatretus burgeri*) as well as the spotted gar (*Lepisosteus oculatus*). By examining the neighboring coding genes of orthologous lincRNAs in each species, we discovered that 21 orthologous lincRNAs exhibited the same neighboring coding genes between *A. polyacanthus* and at least one of the 34 fish species (Table S9). Interestingly, despite having the largest number of orthologous lincRNAs shared with *A. polyacanthus*, the Nile tilapia (*Oreochromis niloticus*) only had four orthologous lincRNAs with the same neighboring coding genes as *A. polyacanthus* (IF4E3: eukaryotic translation initiation factor 4E type 3; SOX8: transcription factor Sox‐8; PIM1: serine/threonine‐protein kinase pim‐1; GP101: probable G‐protein coupled receptor 101). Additionally, only four orthologous lincRNAs have the same neighboring coding genes (A33: zinc‐binding protein A33; IRX5: Iroquois Homeobox 5; KAD4: adenylate kinase 4, mitochondrial; GP101: probable G‐protein coupled receptor 101) shared between *A. polyacanthus* and two or more fish species. For example, *A. polyacanthus* lincRNA “MSTRG.12633” was orthologous with five fish species (*Betta splendens*, *Nothobranchius furzeri*, *Scophthalmus maximus*, *Gouania willdenowi*, *Denticeps clupeoides*; Table S9) and share the same neighboring coding gene Iroquois Homobox 5 (IRX5). Also, lincRNA “MSTRG.30239” in *A. polyacanthus*, which neighbors zinc‐binding protein A33 (A33), was orthologous with the lincRNAs of seven fish species. However, only two species (*Betta splendens* and *Parambassis ranga*) harbor A33 as the neighboring coding gene.

### 3.3 Gene expression changes in fish exposed to ocean acidification

Fish from the CO2 seep displayed a distinct brain expression pattern, both when the analysis was run using all genes (coding genes and lincRNAs; Figure 3b) as well as only lincRNAs (Figure 3c). Between *A. polyacanthus* individuals from CO2 seep and control site, 3431 coding genes (Figure S3a) and 97 lincRNAs (Tables 1 and S10) were significantly differentially expressed by DESeq2 v1.34 (Love et al., 2014). WGCNA results showed that three modules were significantly correlated with environmental CO2 levels (“turquoise”, *p* = 0.001, Table S11; “sky‐blue”, *p* = 0.009, Table S12; “dark‐green”, *p* = 0.006, Table S13). Turquoise and sky‐blue modules were negatively correlated with CO_2_ levels and included 329 lincRNAs and 6259 coding genes (Tables 1 and S11), and 26 lincRNAs and 136 coding genes (Tables 1 and S12), respectively. Dark green module was conversely positively correlated with CO2 levels and consisted of six lincRNAs and 231 coding genes (Tables 1 and S13).

**TABLE 1:** Differentially expressed lincRNAs and coding genes identified through differential expression analysis (DESeq2) and weighted gene co‐expression network analysis (WGCNA).

Gene type	Differential expression	Weighted co‐expression network		
		Turquoise	Sky‐blue	Dark‐green
lincRNAs	97	329	26	6
Coding genes	3431	6259	136	231
lincRNAs with neighboring coding gene	13	65	0	0

*Note*: In the “lincRNAs with neighboring coding gene” row are the numbers of lincRNAs for which the neighboring coding genes were also differentially expressed or found in the same co‐expression module.

### 3.4 Cis‐regulatory roles of lincRNAs in response to ocean acidification

**FIGURE 4:**
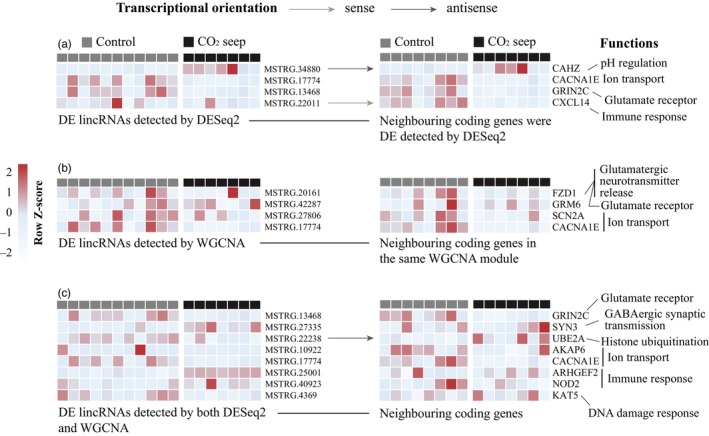
Functions of neighboring coding genes (right) of differentially expressed (DE) lincRNAs (left). (a) DE lincRNAs detected by DESeq2, and their neighboring coding genes were also differentially expressed by DESeq2; (b) DE lincRNAs detected by WGCNA, whose neighboring coding genes were in the same WGCNA module; (c) lincRNAs detected as DE by both DESeq2 and WGCNA and their neighboring coding genes. Heatmaps are created based on normalized reads number by DESeq2 and scaled by row for the z‐score, red means lincRNAs were highly expressed. Black = *Acanthochromis polyacanthus* individuals from CO_2_ seep, grey = individuals from the control site. The arrowed lines signify the transcriptional orientation (sense or antisense) of lincRNAs in relation to their neighboring coding genes, while the absence of an arrow suggests an unknown orientation. The term “sense” refers to a shared transcriptional direction between the lincRNA and its adjacent coding gene, whereas “antisense” denotes an opposing transcriptional orientation.

Based on the significantly differentially expressed (DE) genes detected by DESeq2, DE coding genes with neighboring lincRNAs had significantly higher expression changes (Pearson's chi‐squared test, *p* = 3.425e‐05). 3.5% (7/198) of the DE coding genes with neighboring lincRNA exhibit high expression difference (|log2FoldChange| > 2), whereas only 0.7% (23/3233) of the DE coding genes without neighboring lincRNA display elevated expression (Figure S3b). Among the neighboring coding genes of the 97 DE lincRNAs, 13 coding genes also displayed significant differential expression (Table S14). These DE neighboring coding genes are related to carbon dioxide transport and hypotonic salinity response (CAHZ: carbonic anhydrase), calcium ion transport (CACNA1E: calcium voltage‐gated channel subunit alpha1 E), glutamate‐gated calcium ion channel activity (GRIN2C: glutamate ionotropic receptor NMDA type subunit 2C), or immune system response (CXCL14: C‐X‐C motif chemokine 14; Figure 4a).

Among the significantly correlated co‐expression networks, only the turquoise module was found with 68 lincRNAs whose neighboring coding genes were also included in the same co‐expression module (Tables S11 and S15). The functional implications of these neighboring coding genes show involvement in, for instance, glutamatergic neurotransmitter release (FZD1: frizzled class receptor 1; GRM6: glutamate metabotropic receptor 6) and ion transport (KCNA2: potassium voltage‐gated channel subfamily A member 2; SCN2A: sodium voltage‐gated channel alpha subunit 2; CACNA1E: calcium voltage‐gated channel subunit alpha1 E; Figure 4b).

Among 403 differentially expressed lincRNAs between *A. polyacanthus* individuals from CO2 seep and control site detected by DESeq2 (Table S10) or WGCNA (Tables S11–S13), 52 lincRNAs were significantly differentially expressed detected by both DESeq2 and WGCNA (Table S16). The neighboring coding genes of these lincRNAs were related to the regulation of synaptic plasticity (GRIN2C: glutamate receptor ionotropic NMDA 2C), modulation on GABAergic synaptic transmission (SYN3: synapsin‐3), histone ubiquitination (UBE2A: ubiquitin‐conjugating enzyme E2 A), regulation of ion transport (AKAP6: A‐kinase anchor protein 6; CACNA1E: calcium voltage‐gated channel subunit alpha1 E; UTRN: utrophin), innate immune response (ARHGEF2: rho guanine nucleotide exchange factor 2; NOD2: nucleotide‐binding oligomerization domain‐containing protein 2) and DNA damage response (KAT5: histone acetyltransferase KAT5; Figure 4c). Of the other two significantly correlated modules, sky‐blue and dark green, only two and one lincRNAs, respectively, were also found to be differentially expressed (Tables S12 and S13).

### 3.5 Regulatory roles of potential trans‐acting lincRNAs in response to ocean acidification

Between DE lincRNAs and DE coding genes, 148 lincRNA‐coding gene pairs exhibited high and significant correlation with |rho| ≥ 0.9 and *p*‐value ≤ 0.01, which include 14 lincRNAs and 129 coding genes. With the co‐expression networks, only the turquoise module revealed 219 lincRNA‐coding gene pairs with high and significant correlation made of 14 lincRNAs and 129 coding genes. In summary, 21 lincRNAs may trans‐regulate 186 coding genes (Table S17), including genes involved in ion transport (KCMA1: calcium‐activated potassium channel subunit alpha‐1; SCN8A: sodium channel type 8 subunit alpha; CACNA1A: voltage‐dependent P Q‐type calcium channel subunit alpha‐1A; RYR3: ryanodine receptor 3; NALCN: sodium leak channel non‐selective), glutamate receptor activity (GRM4: glutamate receptor 4; NMDE1: glutamate receptor NMDA 2A), and immune response (BCL11A: B‐cell lymphoma leukemia 11A; BCL11B: B‐cell lymphoma leukemia 11B; BCL9: B‐cell CLL lymphoma 9).

## 4 DISCUSSION

### 4.1 LncRNAs identification

The boost in RNA sequencing studies including a large variety of non‐model organisms provides an excellent resource that could be (re‐)used to obtain a more complete picture of lncRNAs regulations on coding genes. Furthermore, the rapid increase in published genomes for non‐model organisms, owing to international initiatives such as the Earth BioGenome Project (EBP), Darwin Tree of Life Project, and ERGA (European Reference Genome Atlas), offers a valuable opportunity to investigate the regulatory roles of long non‐coding RNAs (lncRNAs) in modulating coding gene expression. Previous lncRNA identification studies primarily relied on a single method for coding potential detection (Boltaña et al., 2016; Dettleff et al., 2020; Mu et al., 2016; Paneru et al., 2018; Quan, Chen, et al., 2020; Ren et al., 2020). However, different tools have shown varying performance levels and apply different prediction methods (Schneider et al., 2017; Wucher et al., 2017). In our pipeline, we employed both alignment‐dependent and alignment‐free tools that have demonstrated success in predicting lncRNAs in other species (Duan et al., 2021). Thus, our pipeline allows us to estimate the coding potential of transcripts while minimizing biases introduced by different methods.

We identified a total of 9209 putative lncRNAs, of which 49% were expressed in at least 75% of our *A. polyacanthus* brain samples. However, as poly‐A selected libraries were used for the RNA library preparation in our study, many lncRNAs lacking a poly‐A tail were less likely to be efficiently captured (Guo et al., 2015; Yang, Duff, et al., 2011), probably leading to lower density of lincRNAs in *A. polyacanthus* compared to protein‐coding genes throughout the whole genome. Overall, the majority of lncRNAs we identified throughout the genome of *A. polyacanthus* had relatively low expression. This observation is in accordance with many previous studies that report a typical low expression of lncRNAs across species and tissues (Guo et al., 2020; Hezroni et al., 2015; Necsulea et al., 2014; Quinn et al., 2016), as lncRNAs are constantly submitted to various regulation mechanisms and they are typically short‐lived (Necsulea et al., 2014; Wu et al., 2014). Despite low expression levels, these lncRNAs can be functional since some regulatory mechanisms do not require high concentration of effector molecules (Aprea & Calegari, 2015). However, some lncRNAs can also be expressed at elevated levels and highly expressed intergenic lncRNAs (lincRNAs), for instance, are associated with diseases (Wan et al., 2013; Yang, Zhang, et al., 2011). In the brains of our coral reef fish, we also found some highly expressed lincRNAs with neighboring coding genes involved in fundamental functions such as a GABA_A_ receptor, circadian rhythm genes and genes related to signal transduction (such as DRD1L and ADA2B), important for normal brain function and neuronal activity (Bhat et al., 2010; Logan & McClung, 2019). In humans, rodents, and zebrafish, lncRNAs promote the expression of homeobox transcription factors required for the development of GABAergic neurons Y‐aminobutyric acid (or GABA) which is the main inhibitory neurotransmitter in the vertebrate brain (Feng et al., 2006). Furthermore, lncRNAs have been shown to regulate core circadian rhythm genes in mammals (Mosig & Kojima, 2022). With a substantial fraction of lncRNAs affecting the gene expression of their neighboring coding genes (Engreitz et al., 2016), it is no surprise to see elevated expression in lncRNAs neighboring GABA_A_ receptor and core circadian rhythm genes as these are known to play important roles in the transcriptional response to ocean acidification (Kang et al., 2022; Schunter et al., 2016, 2021). As our RNAseq data is from ocean acidification experiments, this may suggest a regulatory involvement of lncRNAs in these key functions. Hence, our pipeline of lncRNA annotation and expression analysis allows for the discovery of new lncRNAs that may be involved in the response to an environmental change in a wild coral reef fish, but it also recovers conserved lncRNAs found to possibly be involved in essential functions also in other species.

### 4.2 Low conservation of *A. polyacanthus* lncRNAs across fish species

Multiple studies have demonstrated that lncRNAs exhibit low sequence conservation across species (Darbellay & Necsulea, 2020; Hezroni et al., 2015; Necsulea et al., 2014; Pegueroles et al., 2019; Sarropoulos et al., 2019). This characteristic is also apparent in *A. polyacanthus* lincRNAs with only 6.97% being orthologous to other fish species. Few lincRNAs have conserved sequences and also share the same neighboring coding genes. For example, the orthologous lincRNA between *A. polyacanthus* and five fish species shared the same neighboring coding gene Iroquois Homeobox 5 (IRX5), which is a conserved transcription factor related to energy metabolism and development in vertebrates (Bjune et al., 2019; Bürglin & Affolter, 2016). However, merely four lincRNAs were observed with conserved sequences and same neighboring coding genes across *A. polyacanthus* and at least two other fish species (Table S9). Conversely, the *A. polyacanthus* lincRNAs with distinct sequences comprise the majority of identified lincRNAs, which harbor neighboring coding genes crucial for the responses to environmental changes. For example, we discovered two lincRNAs located adjacent to a heat shock protein (HSP30: heat shock 30), and one lincRNA neighboring the immediate early response gene 2 (IER2), which are both known for their regulatory roles in thermal responses (Chen et al., 2018, 2020; Sandoval‐Castillo et al., 2020). In addition, two lincRNAs were adjacent to V‐type proton ATPases (VAS1: V‐type proton ATPase subunit S1; VTC1A: V‐type proton ATPase subunit C 1‐A), which are responsible for maintaining optimal intracellular pH under varying environmental conditions (Beyenbach & Wieczorek, 2006; Vasanthakumar & Rubinstein, 2020). Notably, 32 lincRNAs were closely located to 23 NLR family CARD domain‐containing protein 3 (NLRC3) genes, which belong to a pathogen recognition receptor family that could negatively regulate immune signals (Schneider et al., 2012; Zhang et al., 2014). With large reductions in NLRC3 genes, a coral reef fish the bluestreak cleaner wrasse (*Labroides dimidiatus*) has evolved a robust immunity to handle the high parasitism environment in coral reef habitats (Kang et al., 2023). Consequently, while *A. polyacanthus* lincRNAs are largely species‐specific with low conservation, they may provide *A. polyacanthus* with specialized regulations to cope with environmental changes.

### 4.3 Functional responses of lncRNAs to ocean acidification

In our study, we made use of a published RNA sequencing set of *A. polyacanthus* brains from a volcanic CO_2_ seep, representing future ocean acidification conditions, and a control site. We detected differentially expressed lincRNAs and mRNAs in the brain of *A. polyacanthus* as a response to natural environmental differences (CO_2_ levels). Interestingly, for the coding genes that have a neighboring lincRNA in close proximity, we found larger expression differences between fish from CO_2_ seeps and control sites suggesting a potential regulation of gene expression by neighboring lncRNAs with the exposure to elevated CO_2_. For the differentially expressed lincRNAs, the set of neighboring protein‐coding genes that were also differentially expressed are involved in functions related to pH regulation in fish. When fish respond to ocean acidification, carbonic anhydrase (CAHZ) is often upregulated, as in our data here, to catalyze the hydration of CO_2_ and play an essential role in acid–base and ion regulatory functions (Heuer & Grosell, 2014). A further reduction of intracellular pH in fish is necessary to prevent acidosis when faced with elevated CO_2_ levels in fish (Schmidt, 2019), and this process performed by coding gene GRIN2C may potentially be regulated by differentially expressed lincRNAs.

Further biological processes are known to be involved in the response to ocean acidification in fish brains. One of them is synaptic transmission and the downregulation of a lincRNA and the neighboring coding gene CACNA1E (voltage‐gated calcium channel complex) can contribute to synaptic transmission (Berecki et al., 2014), which was also found to behave similarly to a previous RNAseq *A. polyacanthus* data (Schunter et al., 2018). Some lincRNAs may trans‐regulate the expression of ion transporters, such as calcium‐activated potassium channel subunit alpha‐1 (KCMA1), sodium leak channel non‐selective (NALCN), and voltage‐dependent P Q‐type calcium channel subunit alpha‐1A (CACNA1A), which play critical roles in the neural signal transduction (Gadsby, 2009) and were also reported in our previous study (Kang et al., 2022). In addition, immune responses are commonly associated with ocean acidification in fish including our study species (Bresolin de Souza et al., 2014; Kang et al., 2022; Machado et al., 2020), and here we find differential expression of lincRNAs neighboring and possibly trans‐regulating a variety of immune response genes that show co‐expression in a gene network significantly correlated with CO_2_ levels. This suggests that lincRNAs annotated in our coral reef fish show differential expression in response to an environmental factor, CO_2_ levels, and that some of these differentially expressed lincRNAs are in close proximity to coding genes that are known to have functional implications in the response to ocean acidification in fishes.

In summary, we employed a comprehensive and effective method, combining alignment‐dependent and alignment‐free tools, to identify candidate lincRNAs in *A. polyacanthus*, revealing the potential involvement in acclimation to elevated CO_2_ levels. We find that the majority of lncRNAs in *A. polyacanthus* are species‐specific, which may enable the species unique adaptive strategies to cope with environmental stressors. Understanding the roles of lncRNAs is critical to explore adaptive potential, providing valuable insights into the molecular mechanisms underlying responses to changing environments. Our study is a start to understand regulatory elements potentially involved in the regulation of the gene expression patterns observed with an environmental change, paving the way for advancements in fields such as evolution, adaptation, and environmental sciences.

## FUNDING INFORMATION

JK, SS, AC, N P‐M and JS were supported through the HKU start‐up to CS. DR was supported by a Hong Kong PhD fellowship. SR was funded by the Research Grants Council of Hong Kong SAR Early Career Scheme fund 27107919 (CS). LB and the project were funded by the General Research Fund from the Research Grants Council of Hong Kong SAR 17300721 (CS). CP is a member of the research group SGR2021‐01271 funded by the Generalitat de Catalunya and CP and CS were supported by MarGeCh (PID2020‐118550RB, funded by MCIN/AEI/10.13039/501100011033) from the Spanish Government.

## CONFLICT OF INTEREST STATEMENT

All authors declare that they have no competing interests.

## CODE AVALABILITY

The scripts have been deposited in Github (https://github.com/jinglkang/lncRNAs_detect).

## Supporting information


**Data S1.** Supporting Information.

**Data S2.** Supporting Information.

## Data Availability

The RNA sequencing raw data used in this study are found in the Bioprojects: PRJNA691990; PRJNA311159; PRJNA658203 (Reviewer link as the last is not published yet: https://dataview.ncbi.nlm.nih.gov/object/PRJNA658203?reviewer=47r5c4kubkjn124c770t5vvt60). The lncRNA annotation of *Acanthochromis polyacanthus* is available here: 10.6084/m9.figshare.20045780. Reviewer link: https://figshare.com/s/33802daaa28dc7ccd876.
