# Supplementary material for: Long non‐coding RNAs mediate fish gene expression in response to ocean acidification
Source: Evol Appl. 2024 Feb 14;17(2):e13655. doi: 10.1111/eva.13655 (PMC10866067; doi:10.1111/eva.13655)
Supplement: Supplementary file 1 — Data S1. Supporting Information. [file EVA-17-e13655-s001.docx]

**Long non-coding RNAs mediate fish gene expression in response to ocean acidification**

Jingliang Kang^1^, Arthur Chung^1^, Sneha Suresh^1^, Lucrezia C. Bonzi^1^, Jade M. Sourisse^1^, Sandra Ramirez-Calero^1^, Daniele Romeo^1^, Natalia Petit-Marty^1^, Cinta Pegueroles^2^, Celia Schunter^1,3*^

^1^Swire Institute of Marine Science, School of Biological Sciences, The University of Hong Kong, Pokfulam Road, Hong Kong SAR

^2^Department of Genetics, Microbiology and Statistics, Institute for Research on Biodiversity (IRBio), University of Barcelona, Barcelona, Spain

^3^ State Key Laboratory of Marine Pollution, City University of Hong Kong, Hong Kong SAR, China

*Correspondence: celiaschunter@gmail.com

SUPPLEMENTARY FIGURES

Figure S1. Sequence features of intergenic long non-coding RNAs (lincRNAs) in *Acanthochromis polyacanthus*. A. Length distributions of lincRNAs. B. Distributions of the number of exons in lincRNAs. C. The GC content in coding genes and lincRNAs. The GC content of lincRNAs were significantly lower (two-sample wilcoxon rank sum test, p < 2.2e-16) than coding genes.

Figure S2. The orientation of intergenic long non-coding RNAs (lincRNAs) in *Acanthochromis polyacanthus.*

Figure S3. Expression pattern comparison between coding genes with (blue) and without (orange) neighbouring lincRNAs. A. Volcano plot of all expressed coding genes. The gene expression log2 fold change and log10(basemean) between samples from CO_2_ seep and control site are reported on the X and the Y axes, respectively. B. Density of log2FoldChange absolute value of gene expression between samples from CO_2_ seep and control site.
